# Using genomics to understand meticillin- and vancomycin-resistant *Staphylococcus aureus* infections

**DOI:** 10.1099/mgen.0.000324

**Published:** 2020-01-08

**Authors:** Stefano G. Giulieri, Steven Y. C. Tong, Deborah A. Williamson

**Affiliations:** ^1^​ Department of Microbiology and Immunology, University of Melbourne at the Peter Doherty Institute for Infection and Immunity, Melbourne, Australia; ^2^​ Infectious Disease Department, Austin Health, Melbourne, Australia; ^3^​ Victorian Infectious Disease Service, Royal Melbourne Hospital, and Doherty Department University of Melbourne, at the Peter Doherty Institute for Infection and Immunity, Victoria, Australia; ^4^​ Menzies School of Health Research, Darwin, Australia; ^5^​ Microbiological Diagnostic Unit Public Health Laboratory, University of Melbourne at the Peter Doherty Institute of Infection and Immunity, Melbourne, Australia; ^6^​ Microbiology, Royal Melbourne Hospital, Melbourne, Australia

**Keywords:** *Staphylococcus aureus*, genomics, antibiotic resistance, MRSA, vancomycin

## Abstract

Resistance to meticillin and vancomycin in *Staphylococcus aureus* significantly complicates the management of severe infections like bacteraemia, endocarditis or osteomyelitis. Here, we review the molecular mechanisms and genomic epidemiology of resistance to these agents, with a focus on how genomics has provided insights into the emergence and evolution of major meticillin-resistant *S. aureus* clones. We also provide insights on the use of bacterial whole-genome sequencing to inform management of *S. aureus* infections and for control of transmission at the hospital and in the community.

## Data Summary

**Fig. 1.**
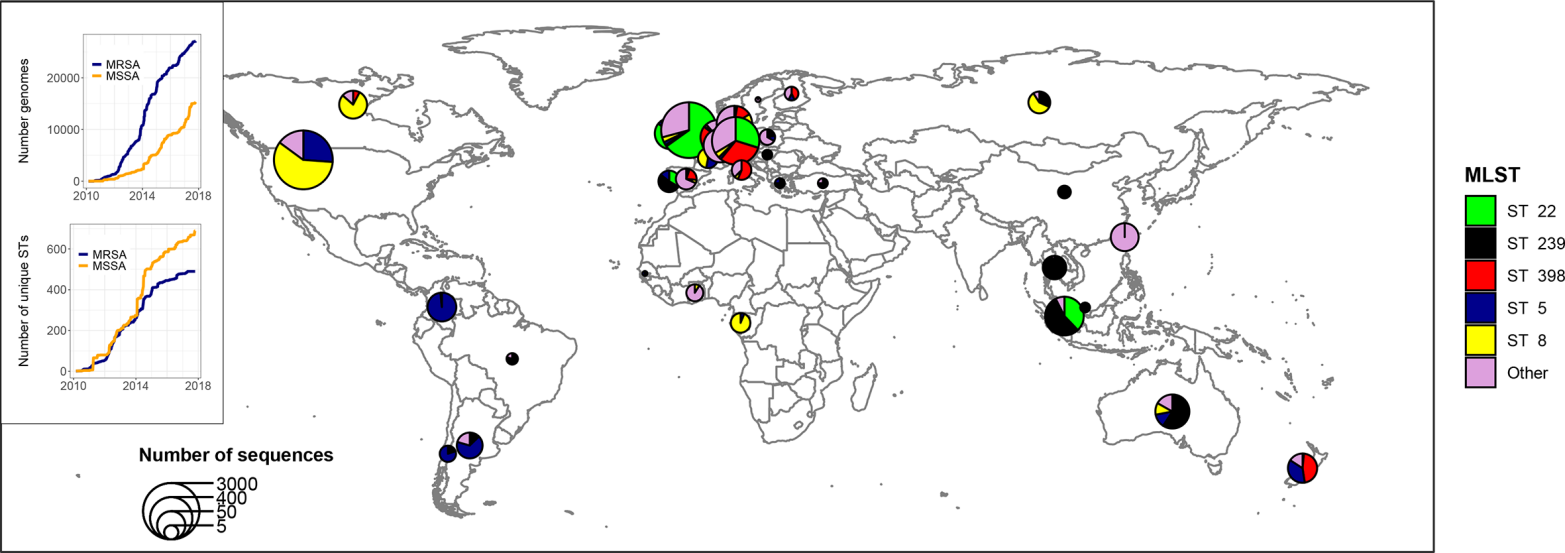
Snapshot of the genomic epidemiology of MRSA based on 42 948 publicly available *S. aureus* genomes (27 120 *mec*-positive) processed through the Staphopia platform (https://staphopia.emory.edu/). The inset shows the explosion of sequenced genomes and the constant increase in genetic diversity with 1099 different STs found in Staphopia. Despite this diversity, 70 % of MRSA sequences belonged to five STs (ST22, ST8, ST5, ST239 and ST398). It should be noted that publicly available *S. aureus* genomics do not accurately represent *S. aureus* epidemiology at this stage, due to sequencing and availability bias and lack of metadata in a large proportion of the dataset.

Fig. 1 was generated using genomic data and geographical metadata extracted from the Staphopia platform (https://staphopia.emory.edu/) using the Staphopia R package (https://github.com/staphopia/staphopia-r).

**Impact Statement**

Meticillin- and vancomycin-resistant *Staphylococcus aureus* have been included by the World Health Organization in the global priority list of antibiotic-resistant bacteria, given the high mortality and morbidity associated with invasive *S. aureus* infections such as endocarditis and osteomyelitis, and the suboptimal outcome of treatment when anti-staphylococcal β-lactams are not available. Whole-genome sequencing (WGS) studies have not only highlighted how meticillin-resistant *S. aureus* spreads in the community and at the hospital, but also shown how use of antibiotics and biocides in the community initiates and amplifies the establishment of drug-resistant *S. aureus *. Moreover, emerging resistance to last-line antibiotics like vancomycin, daptomycin and linezolid can now be dissected at the molecular level by genomic studies. Through increased understanding of the genomic basis of resistance and emerging work on *S. aureus* virulence and persistence, there is likely to be a growing role of WGS in the direct clinical management of *S. aureus* infections.

## Introduction

The facultative pathogen *Staphylococcus aureus* is associated with asymptomatic carriage in 25 % of adults [1] and with a wide spectrum of clinical conditions ranging from skin and soft tissue infections, through to invasive infections such as pneumonia, bacteraemia, infective endocarditis, septic arthritis and osteomyelitis [2]. Invasive *S. aureus* infections still carry a high mortality (for example around 20 and 10 % for endocarditis [3, 4] and pneumonia [5], respectively) and their management can be very complex, particularly when complicated by antimicrobial resistance [6].

The clinical introduction of penicillin in the 1940s dramatically improved the outcome of *S. aureus* infections (the mortality of *S. aureus* bacteraemia in the pre-antibiotic era was as high as 80 %); however, after the introduction of penicillin, resistance spread rapidly, and by 1948 more than the half of tested isolates in one centre were resistant to penicillin [7]. Interestingly, the rise of penicillin-resistant *S. aureus* was subsequently found to be linked to the spread of a single clone, phage type 80/81 [8], the first example of the ‘epidemic waves’ that now characterize the molecular epidemiology of resistant *S. aureus* [9]. A similar phenomenon was observed after the introduction of penicillinase-resistant penicillins (e.g. meticillin, oxacillin) in 1959. Two years later, a report described three clinical *S. aureus* isolates that were resistant to this newly introduced anti-staphylococcal antibiotic [10]. Recent work has established that meticillin-resistant *S. aureus* (MRSA) was already circulating prior to the introduction of meticillin and was likely selected for by penicillin [11]. MRSA subsequently disseminated in the hospital environment, and then separate epidemic waves occurred in the community. By contrast, resistance to the last-line antibiotic vancomycin has developed slowly following its introduction in 1958, with the first report of vancomycin-intermediate *S. aureus* (VISA) by Hiramatsu *et al.* in 1997 [12]. The relatively late emergence of vancomycin resistance was probably related to limited use of vancomycin until the 1980s, when the surge in MRSA infections boosted its use [13]. Resistance to the most recently introduced anti-staphylococcal antibiotics (daptomycin and linezolid) has also been readily acquired: for example, secondary resistance under treatment was described in the randomized controlled trial that led to FDA (Food and Drug Administration, USA) approval of daptomycin [14]; and linezolid resistance, albeit rare, has been reported in series of isolates [15].

In this mini-review, we provide an overview of the major genomic-based insights into the two major clinically relevant mechanisms of staphylococcal resistance (resistance to meticillin and vancomycin), and highlight the contribution of genomic epidemiology to the understanding of the establishment and spread of resistant clones (especially MRSA). Finally, we provide an outline for the future use of genomics beyond resistance research and epidemiology, towards improved individual patient management of invasive *S. aureus* infections, by prediction of antibiotic response, persistence and virulence.

## Genomic insights into MRSA

### Genetic basis of meticillin resistance

*S. aureus* acquires resistance to anti-staphylococcal penicillins through expression of an additional penicillin-binding protein (PBP) (PBP2a) [16]. Unlike other PBPs, PBP2a is resistant to the inhibitory effects of all β-lactams (with the exception of ceftaroline and ceftobiprole) and is almost always encoded by the accessory gene *mecA* [17]. The expression of *mecA* is inducible and controlled by a signal-inducer protein and a repressor located within the *mecA* operon [17]. Accordingly, most MRSA strains express PBP2a at low level, but harbour highly resistant subpopulations (heteroresistance) [18]. High-level resistance can be expressed in special circumstances. An example is the stringent response, i.e. the intracellular accumulation of the second messenger (p)ppGpp secondary to nutritional stress [19, 20]. *In vitro* studies identified genes involved in the stringent response (such as *relA* *)* as ‘auxiliary genes’ that alter the expression of oxacillin resistance, along with several other determinants including the *fem* (factors essential for meticillin resistance) genes [21, 22].

Recently, alternative *mec* alleles have been described. For example, *mecC* shares 70 % nucleotide identity with *mecA* and is typically found in livestock-associated MRSA (LA-MRSA) [23]. Based on two reviews of epidemiological studies [24, 25], this *mec* variant appears to be infrequent and restricted to Europe (with one single case report from Australia [26]). Interestingly*, mecC*-MRSA appears to have a low oxacillin minimum inhibitory concentration (MIC) due to the distinctive characteristics of its PBP2a-homologue (PBP2c), including higher binding affinity for oxacillin than cefoxitin [27], and susceptibility to penicillin-β-lactam inhibitor combinations [28]. Accordingly, *mecC*-MRSA was successfully treated with β-lactams in an experimental endocarditis model [29]. Rarer *mec* homologues are also reported in other staphylococci or related species, such as *mecA1* (* Staphylococcus sciuri *), *mecA2* (* Staphylococcus vitulinus *) or *mecB* and *mecD* (* Macrococcus* spp.) [30–32]. Based on genomic studies, it is hypothesized that *mecA* was acquired several years prior to the first clinical detection of MRSA in 1961. Harkins *et al*. applied a Bayesian phylogenetic inference to a collection of early MRSA isolates (1960–1980) and concluded that meticillin resistance emerged in the mid-1940s, suggesting that the introduction of penicillin may have contributed to the selective pressure that lead to the advent of MRSA [19].

Horizontal transfer of *mecA* is made possible by carriage on a specific mobile genetic element (MGE) ranging between 23 and 68 kb in size, the staphylococcal cassette chromosome (SCC) [33]. The association of *mecA* with the SCC (termed the SCC*mec*) not only is important for *mecA* acquisition or transfer, but also is a key factor mediating antimicrobial co-resistance, since genes conferring resistance to non-β-lactam antibiotics can be co-located in the same locus [34–36]. To date, 13 variants of SCC*mec* have been described [37], but with the increasing number of sequenced strains, new variants are likely to be discovered in the future. Beyond SCC*mec*, other MGEs are critical for acquisition and dissemination of resistance to antibiotics and biocides (particularly plasmids and transposons, see the review by Firth and colleagues [38]) and virulence determinants (particularly bacteriophages, see the review by Xia and Wolz [39]).

The conserved structure of SCC*mec* and *mecA* has facilitated the molecular detection of meticillin resistance; molecular point-of-care tests have streamlined the rapid detection of MRSA from clinical samples. However, correlation between the presence of *mecA* or *mecC* and phenotypic resistance to oxacillin is not absolute – approximately 3 % of *S. aureus* strains harbouring *mecA* are phenotypically susceptible to oxacillin [40, 41]. As mentioned above, this phenomenon has been previously explained by heterogeneous synthesis of PBP2a [42], but a genomic study provided an interesting alternative mechanism. The authors investigated two clinical isolates of *mecA*-positive meticillin-susceptible *S. aureus* (MSSA) and demonstrated that *mecA* expression was suppressed by disruption of the gene through insertion of IS*1181* in one case and a *mecA* frameshift mutation in the other [41]. β-Lactam sensitivity in MRSA has been investigated in a recently published study by Harrison *et al*., where they identified a subset of MRSA strains that were susceptible to penicillin/clavulanic acid combinations. The genomic basis of this phenomenon was found to be the association of mutations both in the promoter and coding sequence of *mecA* [43].

Conversely, oxacillin resistance can be mediated by other mechanisms than PBP2a production. Such *mecA-* (and *mecC*)-negative, oxacillin-resistant strains [borderline oxacillin-resistant *S. aureus* (BORSA)] are increasingly recognized and may be associated with failure of oxacillin clinical therapy, typically in complex, deep-seated infections [44, 45]. While previous work has investigated β-lactamase hyperproduction or PBP mutations [46], recent genomic studies of BORSA isolates have linked this phenotype to mutations in the regulatory gene *gdpP* [47, 48], which degrades the second messenger c-di-AMP. *gdpP* mutations have been linked to changes in cell-wall metabolism and increased resistance to antibiotics targeting the cell wall like β-lactams and vancomycin [49].

### Genomic epidemiology of MRSA

The genomic epidemiology of MRSA is multifaceted. MRSA is typically clonal, with epidemic waves of infections characterized by the temporal rise and decline of clones [50, 51]. Parallel to these chronological changes, geographical segregation can be observed, with some adaptation to specific environments, including health-care facilities, community settings and animal populations. These broad groupings form the basis for the often used classifications of health-care-associated MRSA (HA-MRSA), community-associated MRSA (CA-MRSA) and livestock-associated MRSA (LA-MRSA). Previously, molecular epidemiology using lower-resolution approaches, such as pulsed-field gel electrophoresis (PFGE), multilocus sequence typing (MLST) or *spa* typing, helped delineate dominant MRSA clones and track their emergence, expansion and spread [52]; however, over the past 10 years, whole-genome sequencing (WGS)-based studies have defined the complex epidemiology of MRSA, from tracking global dissemination of successful clones, to dissecting chains of transmission in hospitals and the community, and between livestock and humans.

Harris *et al*. performed the first study that applied large-scale bacterial WGS to explore global dissemination of the HA-MRSA clone sequence type (ST)239 [53]. While the core-genome phylogeny was consistent with PFGE and *spa* typing, genomic data provided detailed insights into the phylogeographical structure of the ST239 lineage, the emergence of antibiotic-resistance mutations and transmission within a health-care facility. The impact of genomics on the investigation and control of hospital-associated MRSA outbreaks has also been demonstrated in subsequent papers [54–56], while others have analysed MRSA transmission networks in the community setting [57] or described the emergence of LA-MRSA and the complex interplay associated with transmission from humans to farm animals and vice versa [58, 59]. Table 1 provides a selection of the major genomic epidemiological studies associated with MRSA and their key findings. Given the large number of studies that have applied WGS to address MRSA epidemiology, we have selected studies based on (i) their novelty at the time they were published, and (ii) the range of MRSA genomic epidemiology (e.g. global or local transmission, adaptive evolution, source attribution in LA-MRSA).

**Table 1.** Selection of studies that used WGS to investigate the genomic epidemiology of MRSA

Setting	Publication	MRSA clone	No. of isolates (timeframe)	Main findings	Rationale for inclusion
I**nternational studies**					
Health-care associated	Harris *et al.*, *Science* 2010 [53]	ST239	63 (1982–2007)	Phylogeographical analysis highlighting global dissemination of ST239 HA-MRSA and transmission within one hospital in Thailand. Comparison to *spa* typing and PFGE shows that WGS has higher resolution.	First genomic epidemiology study of *S. aureu* *s *.
Livestock associated	Price *et al.*, *mBio* 2012 [58]	ST398	89 (1993–2010)	Worldwide genomic study of ST398 revealing that the ancestral clades were human-associated and meticillin susceptible, and that after human to livestock transition the clone underwent host adaptation. Adaptation to livestock was marked by acquisition of meticillin and tetracycline resistance, and loss of the phage carrying human-associated immune evasion cluster (IEC).	First genomic description of the LA-MRSA clone ST398.
Health-care associated	Holden *et al.*, *Genome Res* 2013 [69]	ST22 (EMRSA-15)	193 (1990–2009)	Detailed reconstruction of the evolution of the ST22 clone using Bayesian methods showed: (i) dissemination first throughout the UK and then globally, and (ii) progressive acquisition of antibiotic-resistance determinants (SCC*mec* IV and *grlA* and *gyrA* mutations associated with fluoroquinolone resistance). Acquisition of resistance to β-lactams and fluoroquinolones has likely contributed to the success of the clones, leading to an expansion of the subclade ST22-A2.	An in-depth genomic study of the emerging HA-MRSA clone ST22 using a Bayesian framework to decipher genetic determinants of clonal dissemination.
Multiple settings	Aanensen *et al.*, *mBio* 2016 [60]	Multiple STs	308 (2006–2007)	Genomic snapshot of invasive *S. aureus* isolates collected across 186 hospitals in Europe. This study provided a framework to study genomic epidemiology of *S. aureus* at a continental level. It showed high genetic diversity, especially among MSSA. MRSA clones (but not MSSA) were geographically clustered with evidence of international transmission.	This study showed the potential use of genomics for surveillance of *S. aureus* epidemiology at continental scale.
Community associated	Ward *et al*, *Genome Biol* 2016 [76]	ST59	120 (1998–2011)	Global study of CA-MRSA clone ST59 that is dominant in East Asia. Two distinct clades were identified that emerged in the USA and in Taiwan. Transitions between countries were mapped using a ‘Markov jumps’ method based on a Bayesian phylogenetic approach. This analysis identified Taiwan and the USA as ‘source countries’ as opposed to the ‘sink countries’ the UK, Australia and the Netherlands.	Innovative use of Bayesian methods to track transmission between countries.
Community associated	Van Hal *et al.*, *Front Microbiol* 2018 [73]	ST93	459 (1991–2012)	Global study of CA-MRSA clone ST93 that determined ST93-MRSA emerged from indigenous communities in northern Australia and subsequently spread to the east coast of Australia, New Zealand and Europe. A locally adapted clone to NZ was evident.	Example of global dissemination of a CA-MRSA clone.
Community associated	Steinig *et al.*, *mBio* 2019 [159]	ST772	340 (2004–2013)	Global study of CA-MRSA clone ST772 that determined ST772 emerged from the Indian subcontinent in the 1970s with sporadic transmission overseas. Acquisition of a multidrug-resistance plasmid was instrumental in the emergence and dissemination of a globally disseminated clade in the 1990s.	Genomic epidemiological study applied to an emergent community-associated clone from South Asia
**Country-wide studies**					
Community associated	Stinear *et al.*, *Genome Biol Evol* 2014 [71]	ST93	56 (2000–2009)	Comprehensive study of the adaptive evolution of the Australian CA-MRSA clone ST93, showing progressive reduction in secretion of exotoxins and decrease in oxacillin MIC.	Example of an in-depth study combining genomics and phenotypic analysis to study adaptive evolution.
Health-care associated	Baines *et al.*, *mBio* 2015 [70]	ST239	123 (1980–2012)	Genomic and phenotypic study of the adaptive evolution of the hospital-adapted MRSA clone ST239 in Australia. The analysis showed that adaptive changes (virulence attenuation and increased antibiotic resistance) and the introduction of a previously undetected ST239 clade from Asia promoted ST239 persistence in the hospital environment.	Another example of adaptive evolution study applied to a hospital-associated clone.
Community associated	Baines *et al.*, *Antimicrob Agent Chemother* 2016 [34]	ST1	46 (2005–2013)	Genomic investigation of the expansion of the fusidic acid-resistant CA-MRSA ST5 clone in New Zealand, which was associated with increased use of topical fusidic acid. The key finding was the consistent co-location of the fusidic acid-resistance determinant *fusC* with the SCC. This study indicates that topic antimicrobials can drive the emergence of MRSA through co-resistance and co-location of resistance genes.	Genomic demonstration of the role of co-resistance in promoting the emergence of CA-MRSA.
Health-care associated	Reuter *et al.*, *Genome Res* 2016 [61]	Multiple STs	1013 (2001–2010)	Large genomic surveillance study of MRSA bloodstream infections in the UK and Ireland used to infer transmission and resistance acquisition. The study showed a dominance of ST22 (EMRSA-15), and ST36 (EMRSA-16) and phylogeographical clustering around referral networks.	First demonstration of systematic WGS of invasive MRSA infections.
Livestock associated	Gonçalves da Silva *et al.*, *Microb Genom* 2017 [59]	ST398	147 (2004–2015)	This study investigated the emergence of the LA-MRSA clone ST398 in New Zealand. A Bayesian phylogenetic approach was used to infer that the clone originated in swine from western Europe.	Example of use of Bayesian phylogenomics for source attribution in LA-MRSA.
Mixed setting	Coll *et al.*, *Sci Transl Med* 2017 [64]	ST22 and others	1465 (2012–2013)	Genomic investigation of MRSA transmission within a single hospital referral network in the UK. The study revealed several clusters of transmission both at the hospital and community (general practice) level.	Powerful demonstration of the use of integrated genomic and epidemiological data (extracted from hospital administration, patient postcodes, general practice attendance) for MRSA transmission surveillance.
**Single institution studies** **/** **communities**					
Hospital associated	Köser *et al*., *N Engl J Med* 2012 [54]	ST22	14 (2009)	Demonstration of the use of rapid WGS to investigate and manage a MRSA outbreak in a neonatal intensive care unit in the UK.	First use of WGS to track hospital transmission of MRSA.
Community associated	Uhlemann *et al.*, *Proc Natl Acad Sci USA* 2014 [57]	ST8 (USA300)	378 (2009–2011)	Genomic investigation of transmission networks of ST8 CA-MRSA (USA300) within a New York community, showing that community households played a major role in maintaining and disseminating carriage and infection.	This study used WGS to track transmission of USA300 in the community.
Health-care associated	Tong *et al.*, *Gen Research* 2015 [75]	ST239	79 (2008)	Investigation of endemic MRSA in a developing world hospital setting that demonstrated considerable diversity within two intensive care units in a Thailand hospital. Also demonstrated that individuals can be colonized by a cloud of strains.	First genomic description of MRSA in a developing world hospital setting.
Health-care associated	Senn *et al.*, *mBio* 2016 [55]	ST228	228 (2008–2012)	WGS study investigation of a sustained MRSA outbreak in a Swiss teaching hospital. Genomic and epidemiological data revealed that the outbreak was due to a single ST228 clone. Low genetic diversity within the isolates suggested direct transmission between patients, possibly due to increased transmissibility and to more frequent rectal carriage, as compared to other clones.	This in-depth genomic investigation of a single-institution MRSA outbreak showed that genomics can be used to identify hidden MRSA reservoirs and investigate transmission dynamics.
Hospital associated	Price *et al.*, *Lancet Infect Dis* 2017 [63]	Multiple STs	1819 (2011–2012)	Comprehensive longitudinal sampling of *S. aureus* strains from patients, a health-care worker and the environment in a single-centre intensive-care unit. Genomics showed constant introduction of novel subtypes, with infrequent transmission to patients.	First use of WGS to track *S. aureus* transmission between patients, a health-care worker and the environment.

More recent *S. aureus* genomic epidemiological studies have evolved in two complementary directions: (i) expanding the breadth of the analysis by providing local, national or international surveillance frameworks for MRSA (and *S. aureus* in general); or (ii) performing in-depth investigations of single clones or lineages, and exploring the interplay between adaptive evolution and antibiotic pressure. For example, Aanansen *et al*. performed a population genomic study of 308 *S *. *aureus* isolates across 21 European countries. Their data provided a ‘snapshot’ view of the genetic diversity of *S. aureus* across a continent, and allowed the investigation of evolution within single clones and intercontinental transmission, as well as identification of both MSSA and MRSA ‘high-risk clones’ (e.g. CC22, CC30) based on speed of expansion as assessed from phylogenomics, phylogeographical structure and the presence of key resistance or virulence genes. Their study also showed that the population structures of MRSA and MSSA are fundamentally different, with the former being more clonal and geographically clustered [60]. Building on a similar approach, Reuter *et al*. described the population structure of over 1000 invasive MRSA isolates from the UK and Ireland [61]. An important finding of their study was strong phylogeographical clustering around hospital referral networks, highlighting the potential for the use of WGS in epidemiological surveillance and early identification of new hospital outbreaks [61, 62]. Two recent studies expanded the framework of surveillance of MRSA transmission: Price *et al*. showed that genomics can be applied to interrogate the complex transmission interplay between patients, health-care workers and the environment in the health-care setting [63], while a study by Coll *et al*. integrated genomic data with epidemiological data retrieved from various sources (hospital admissions, postcodes, general practice attendance) to reconstruct MRSA transmission networks both in the hospital and the community [64].

With over 40 000 *S *. *aureus* genomes now publicly available, large-scale genomic surveillance is now possible [65, 66]. Fig. 1 demonstrates the global distribution of MRSA clones based on publicly available *S. aureus* genomes processed through the Staphopia platform [65]. Despite the great interest of this large and ever-growing public dataset of *S. aureus* sequences, it should be noted that these data are not necessarily representative of the actual *S. aureus* epidemiology (sources of bias include the larger availability of sequencing in a small group of developed countries, increased sequencing of MRSA for public-health reasons and lack of metadata including country of collection for a large proportion of the isolates). Other available genomic platforms for *S. aureus* (and other bacteria) offer access to publicly available genomes and allow comparison of sequences uploaded by the user through analysis pipelines, e.g. patric (https://www.patricbrc.org), National Center for Biotechnology Information (NCBI) Pathogen detection (https://www.ncbi.nlm.nih.gov/pathogens/; however, *S. aureus* is not included yet) and Pathogenwatch (https://pathogen.watch). These repositories and new computational approaches allow high-throughput analysis of stored sequence data for both rapid and efficient genomic surveillance [67] and discovery of genetic determinants of resistance or pathogenesis [68].

### In-depth genomic studies of specific MRSA lineages

An early example of an in-depth, clone-specific approach is reported in a study by Holden *et al*., who applied Bayesian phylogenetics methods to dissect the evolutionary history of the hospital-associated ST22 clone, the dominant MRSA clone in the UK. The analysis reconstructed the acquisition of important antibiotic-resistance determinants (*mecA* and resistance-associated mutations in the *gyrA* and *grlA* genes) and showed that rapid expansion and dissemination of sublineage ST22-A2 was likely promoted by acquisition of fluoroquinolone resistance [69]. Other authors have combined population genomics and phenotypic studies to dissect adaptive micro-evolution of MRSA both in the hospital [70] and community environment [71–73]. For example, two genomics studies of ST93, a community-acquired MRSA clone in Australia, have revealed how this lineage likely arose in a remote area in North-West Australia and subsequently disseminated across the continent and overseas [71, 73]. One study also showed that this high-virulence clone changed its phenotype towards reduced virulence (e.g. expression of alpha-toxin) and increased susceptibility to oxacillin [71], possibly indicating adaptive evolution to the health-care environment, as previously shown in other clones such as CC30 [74]. Another Australian study explored adaptive evolution of the hospital-associated ST239 clone. Phylogenetic analysis showed that the epidemics had been enhanced by the introduction of a previously unrecognized sublineage from Asia. Both the Australian and the Asian sublineage of ST239 exhibited patterns of convergent evolution, namely decreased virulence and increased resistance to antibiotics (including vancomycin) – both characteristics of hospital adaptation. The transmission potential of ST239 was also highlighted in a study performed in an hospital in Thailand [75]. Further, in a worldwide study of the CA-MRSA clone ST59 (dominant in East Asia), the authors applied a Bayesian phylogenetic method (‘Markov jumps’) to identify ‘source’ countries (USA, Taiwan) and ‘sink’ countries (Australia, the UK, the Netherlands) of the ST59 epidemic [76]. Collectively, these studies have provided clear examples of the utility of population genomics (complemented with relevant phenotypic testing) in understanding the evolutionary mechanisms that underpin the success of MRSA clones.

### Co-resistance in MRSA

An area of ongoing interest is the role of co-resistance to non-β-lactam antibiotics in the spread and expansion of MRSA clones [77]. Co-resistance is of both epidemiological and clinical relevance, since it has been shown that use of selected antibiotics (e.g. fluoroquinolones) may drive the MRSA epidemic [78]; thus, offering potential targets of preventive interventions through antibiotic stewardship in human health or in agriculture [79]. This is not only true for systemic antibiotics, but also for topical antibiotics and biocides [80].

From a genomic perspective, co-resistance can arise via four different mechanisms: (i) the same genetic determinant (gene or mutation) can confer resistance to multiple antibiotics (e.g. the pleiomorphic effects of *rpoB* mutations, which include decreased susceptibility to vancomycin and daptomycin [66]); (ii) compensatory mutations that counterbalance the fitness cost associated with resistance to one antibiotic can alter susceptibility to another drug (e.g. increased β-lactam susceptibility in VISA, see below [81]); (iii) the genetic resistance determinants are co-located on the same mobile element (e.g. within the SCC*mec*) [36]; or (iv) the resistance determinants are co-located within the same strain (e.g. fluoroquinolone resistance in MRSA).

Co-location on the same MGE is important, because it is associated with a risk of horizontal transmission of both genetic determinants. In MRSA, it is enabled by the plasticity of the SCC*mec* element that can host several genes associated with resistance to antibiotics or heavy metals [82]. For example, the erythromycin-resistance gene *erm*(A) is found on transposon Tn*554*, which is in turn nested within type II, III and VIII SCC*mec* elements [33], while the tetracycline-resistance plasmid pT181 is integrated in type III SCC*mec*. Similarly, the aminoglycoside-resistance gene *aacA-aphD* is carried on transposon Tn*4001*, which can in turn be found not only on several multi-resistance plasmids, but also on some SCC*mec* elements [83]. An illustrative example of the effect of resistance co-location on MRSA epidemiology is provided by the rapid emergence of fusidic acid-resistant MRSA and MSSA in New Zealand that was likely fuelled by the unrestricted use of topical fusidic acid. Genomic studies showed that fusidic acid resistance was restricted to two dominant MRSA clones (ST5 and ST1) and one MSSA clone (ST1) that had acquired the fusidic acid-resistance determinant, *fusC*. Crucially, the *fusC* operon was exclusively located in SCC elements, in both MSSA and MRSA [34, 84]. Important MRSA co-resistance determinants located outside SCC*mec* are the quinolone-resistance genes *gyrAB* and *grlAB*, encoding the DNA gyrase and DNA topoisomerase, respectively [85]. Population genomics studies show that a single acquisition of quinolone resistance in the 1990s drove clonal expansion in both ST8 (USA300) [57] and the ST22 lineage (EMRSA-15) [69].

Tolerance to biocides and resistance to topical antibiotics can also be mediated by genes located on plasmids. The *qacA* gene encodes an efflux pump that is associated with tolerance to monovalent and divalent cations such as chlorhexidine, a widely used disinfectant in the hospital setting. It is generally carried on pSK1 family plasmids, often in combination with other resistance genes such as the β-lactamase *blaZ*. A recent adaptive evolution study has shown a progressive decrease of chlorhexidine sensitivity among ST239 isolates. This phenotypic change was associated with a complex rearrangement of the pSK1 plasmids [86]. Mupirocin resistance is linked to mutations in the chromosomal gene *ileRS* (low-level resistance) or the plasmid-located gene *mupA* [87]. A recent genomic study showed that mutations in the essential gene *ileS* appear to have pleiotropic effects [88], highlighting the complexity of antibiotic resistance in *S. aureus *.

### Using genomics to explore virulence in MRSA

The complexity of virulence has been recently highlighted [89]. It has been very difficult to identify single genomic determinants of clinical outcome in *S. aureus* infections [90], with the exception of some toxin-mediated syndromes [91, 92]. Nevertheless, it is possible to classify genetic determinants of virulence based on broad phenotypic characterization in experimental models (e.g. adhesion, toxin production, immune evasion and gene regulation) and their genetic context (i.e. core genome or accessory genome). Although a detailed description of virulence determinants is beyond the scope of this review, we will highlight some recent insights into *S. aureus* virulence that have been specifically provided by genomic studies.

A striking feature of CA-MRSA clones (such as ST8 and ST93) has been increased virulence in animal models and clinical examples of severe diseases (such as necrotizing skin or lung infections) [93, 94]. This has also been demonstrated *in vitro* as increased cytotoxicity against human lymphocytes or macrophages [95]. While the genetic basis of increased virulence remains elusive, these clones were characterized by the presence of the Panton-Valentine leukocidin (PVL) toxin encoded by two genes *lukS* and *lukF*, located on a bacteriophage [96]. There remains controversy around the true clinical relevance of PVL [97]; however, a recent genome-wide association study (GWAS) showed that it was strongly associated with *S. aureus* pyomyositis among children in Cambodia [98]. Further, it has been shown that cell toxicity resulting from exotoxin production in MRSA might be related to regulatory mechanisms rather that a single gene or locus. For example, there is an inverse relationship between PBP2a expression and toxicity; generally, classic CA-MRSA clones such as ST8 and ST93 have a lower oxacillin MIC and higher toxicity [99].

Other virulence determinants identified in genomic studies are the arginine catabolic mobile element (ACME), a large genetic segment possibly enhancing colonization in ST8 MRSA [100] and *sasX,* carried on a prophage in ST239 MRSA [101]. It is expected that genomic studies will continue to identify previously unrecognized virulence determinants. For example, a recent analysis of 92 USA300 isolates from an outbreak in a New York community identified mutations in the pyrimidine nucleotide biosynthetic operon regulator *pyrR* that were associated with enhanced fitness *in vitro* and enhanced colonization and transmission in a mouse model [72]. Furthermore, genomic analysis revealed that the acquisition of a bacteriophage was associated with larger skin abscesses in an animal model, emphasizing the impact of structural variants and MGEs on clone success and staphylococcal pathogenesis [72].

## Genomic insights into vancomycin-resistant *S. aureus*

The first report of vancomycin resistance was published in 1997 [12], 39 years after vancomycin was first introduced [102]. The authors isolated an MRSA strain with a vancomycin MIC of 8 mg l^−1^ from a patient with a persistent sternal wound infection, who had been exposed to vancomycin for several weeks [12]. From a molecular perspective, vancomycin resistance in *S. aureus* can arise through acquisition of the vancomycin-resistance determinant *vanA,* or more commonly via an array of *vanA*-independent mechanisms, mostly mutations in genes involved in cell-wall biosynthesis [103]. *vanA*-mediated resistance is associated with high-level resistance (VRSA, with a vancomycin MIC of 16 mg l^−1^ and higher) and is due to acquisition of the *vanA* operon, located on transposon Tn*1546* [104], more commonly associated with vancomycin resistance in enterococci [105]. It was first described in 2002 in a patient with end-stage renal failure and diabetic foot infection [106]. Subsequent molecular studies demonstrated that the VRSA isolate carried a conjugative plasmid that had acquired Tn*1546* from a co-infecting vancomycin-resistant *Enterococcus faecalis* [104]. While this report and previous experimental work [107] raised concerns of dissemination of high-level vancomycin resistance, VRSA remains very rare, with a total of only 14 cases reported in the USA [108], and a few reports from Iran [109] and India [110]. Although most VRSA strains to date belong to clonal complex 5, genomic analyses of 12 VRSA strains from the USA showed that they were genetically distant, with the most recent common ancestor around 1960 and likely independent acquisition of the plasmid-born *vanA* operon in each isolate [111].

Since its first description in 1997, several studies have investigated the complex background of *vanA*-independent vancomycin resistance. Phenotypically, these strains have low-level vancomycin resistance (VISA, vancomycin MIC 4–8 mg l^−1^) or may not be resistant when tested with conventional methods, yet harbour vancomycin heteroresistance (hVISA) [103]. They are also characterized by a thickened cell wall [112], slower growth and increased autolysis [113]. The molecular basis of these changes is complex and polygenic (extensive reviews have been published by Howden *et al*. [114] and McGuinness *et al*. [108]). Most mutations involve regulators of cell-wall biosynthesis, such as the two-component regulators *vraRS* [115], *graRS* [116] and *walKR* [117]. However, mutations in the *rpoB* gene (with or without co-resistance to rifampicin) [118] and in the PP2C phosphatase *prpC* [119] can also be associated with the VISA phenotype. Interestingly, in one case, decreased vancomycin susceptibility was linked to insertion of the transposon IS*256* upstream of *walKR* [120], possibly altering its expression [121]. To date, two GWASs have assessed putative mutations associated with the VISA phenotype, both on ST239 isolates. The first study of 123 isolates found an association with a SNP in *walkR* [70], while the second (75 isolates) pointed to the H481Y/L/N *rpoB* mutation [122]. Further, a study using a machine-learning approach found that the VISA phenotype could be predicted with 84 % accuracy [123]. Although reduced vancomycin susceptibility can be found in any genetic background [124], ST239 strains tend to have a higher vancomycin MIC [125]. ST5 seems also to be more often associated with VISA [126].

Despite different genetic resistance mechanisms and phenotypes, VRSA and VISA share common features that distinguish them from MRSA. Unlike MRSA, VRSA and VISA are generally polyclonal, and no significant dissemination has been documented. To date, vancomycin resistance has occurred secondarily, during treatment for complicated *S. aureus* infections. As such, prevention of this resistance is likely best achieved through optimising the management of complex MRSA infections (including appropriate source control) and implementing antibiotic stewardship, rather than through infection control. However, there remains an omnipresent risk that vancomycin resistance could disseminate more effectively, especially with widespread transfer and expansion of a *vanA*-harbouring clone [127].

Unfortunately, resistance to newer anti-staphylococcal antibiotics is also emerging. Daptomycin has been proposed as a possible alternative to vancomycin for the treatment of invasive MRSA infections (with the exception of pneumonia) [128]; however, VISA/hVISA are often co-resistant to daptomycin [129] and secondary resistance can develop *in vivo*, especially in the case of deep-seated infections and poor source control [130]. Genetically, the main mechanism of daptomycin resistance is considered to be gain-of-function mutations in *mprF*, which encodes for a lysyltransferase producing lysylphosphatidylglycerol, a positively charged cell-membrane lipid that mediates resistance to host antimicrobial peptides [131]. It is hypothesized that mutations associated with daptomycin resistance increase cell-membrane positivity and, hence, impair binding of daptomycin, which is positively charged [132]. Similar to the VISA phenotype, comparative genomics studies of closely related isolates (either from cases of daptomycin treatment failure or from *in vitro* exposure experiments) have been instrumental in identifying further genetic determinants of daptomycin resistance. Strikingly, some of these genes are the same as those implicated in the VISA phenotype, such as *walKR* [133], *rpoB* [118] or *prpC* [119]. Furthermore, both daptomycin and vancomycin resistance are associated with the ‘see-saw’ effect, where increased resistance to glycopeptides and lipopeptides leads to reduced resistance to β-lactams [81]. The molecular basis of this phenomenon is complex and only partially investigated; for example, some studies have shown compensatory changes in the *mecA* gene [134] and reduced *mecA* expression [135].

Linezolid is a potential alternative anti-MRSA antibiotic that is not known to be affected by co-resistance to vancomycin. Resistance to linezolid can arise through to point mutations in 23S ribosomal RNA [136] and the ribosomal proteins L3/L4 [137]; however, it can also be acquired through transfer of the accessory gene *cfr*, which produces a 23S rRNA methylase [138]. This gene is often carried by a plasmid [139] and a small multi-clonal outbreak of *cfr*-positive MRSA has been described in a Spanish hospital [140]. Ceftaroline, a next-generation cephalosporin with specific activity against PBP2a, might be used either as salvage therapy or as part of combination treatment for invasive MRSA infections [141]. However, ceftaroline resistance has been described in several MRSA lineages, both at baseline and on treatment [142], mainly through point mutations in *mecA* or in *pbp4* [143, 144]. Interestingly, *mecA* polymorphisms associated with ceftaroline resistance were found in a Korean study, despite the fact that ceftaroline had not yet been used in the country [145]. In a study of 421 strains, 17 % were non-susceptible to ceftaroline (>1 mg l^−1^), with a higher proportion in ST239 MRSA [146].

## Applying genomics to the management of invasive *S. aureus* infections

In addition to population-level studies, genomics has been increasingly used in the clinical microbiology laboratory at the patient level, mainly in the prediction of phenotypic resistance from genotypic data. Several translational studies have shown that genomic prediction of antibiotic resistance is highly accurate in the case of *S. aureus* [147, 148]. The main issue with this approach is related to yet unknown resistance mechanisms [149]; however, it is likely that prediction accuracy will further improve as databases of genetic determinants of resistance are constantly updated, provided that careful genotype–phenotype association studies are also performed.

From a clinical perspective, one area where genomics offers considerable potential is through the use of WGS data to predict clinical outcomes and inform patient management, beyond considerations of antimicrobial resistance [90]. Previous molecular studies using multiple PCR or DNA arrays have suggested an association between certain clonal types and clinical manifestations or outcomes of *S. aureus* bacteraemia; however, with a few exceptions [150], no consistent link was demonstrated between the presence/absence of specific genes or mutations and clinical outcomes. More recently, Recker *et al*. used WGS data and applied a machine-learning algorithm to a *S. aureus* bacteraemia cohort in the UK to map associations between bacterial genetics, phenotypes potentially associated with virulence (cytotoxicity and biofilm formation) and clinical outcome [151]. The main finding of the study was that bacterial phenotype and genotype contributed to infection outcome; however, the effect appeared to be clone-specific, highlighting the complexity of outcome predictions in this setting. Another study from Denmark was not able to determine bacterial genomic predictors of infective endocarditis in *S. aureus* bacteraemia, despite using multiple genomic approaches [152]. Prediction might be more straightforward for rarer, specific clinical *S. aureus* syndromes. For example, Young *et al*. successfully applied GWAS to further highlight the role of PVL in the pathogenesis of pyomyositis [98]. However, to provide findings that can be implemented in clinical management, larger studies of genetic determinants of *S. aureus* infection outcomes are needed. Crucially, these studies will require additional validation either in independent cohorts or through functional tests [153], as well as integration of clinical covariables and, ideally, host genomics [154].

An alternative approach to uncover bacterial genetic determinants of disease is to investigate bacterial host adaptation through within-host evolution studies [155]. The study of genetically closely related isolates from the same patient offers a unique opportunity to identify new bacterial molecular markers of resistance, virulence or persistence without the need for large patient cohorts and without the analytical problems associated with GWAS approaches. These studies have played an essential part in establishing the genetic factors underlying VISA [114], but may also offer insights into the pathogenesis of invasive *S. aureus* infections [120, 156]. Furthermore, comparative genomics of multiple patient isolates could help manage treatment failure by a reliable differentiation between true relapse and reinfection, or by the identification of *de novo* resistance mutations, especially if novel techniques are used that allow accurate detection of low-frequency populations [157].

## Conclusion and future directions

*S. aureus* remains a considerable clinical burden, in both hospital and community settings. This is aggravated by resistance to key anti-staphylococcal antibiotics like flucloxacillin and vancomycin. Molecular and genomic studies have provided invaluable insights into how resistance arises. For MRSA, they have demonstrated how MGEs have facilitated the selection and dissemination of distinct clones in hospital wards, community networks and at a global scale. Further, genomic datasets are now available, allowing the prediction of resistance to common antimicrobials, with ongoing work trying to accurately predict genotypic resistance to last-line antibiotics such as vancomycin, daptomycin, linezolid and ceftaroline. Future studies should also investigate whether bacterial genomics can be used to predict antibiotic synergism and response to combination therapy (e.g. vancomycin/daptomycin combination with β-lactams [158]). However, this large amount of genomic information can only be exploited if high-quality metadata are collected and (where possible) made publicly available. For example, phenotypic antibiotic susceptibility should be submitted along with other metadata (an approach encouraged by the NCBI, as described at: https://www.ncbi.nlm.nih.gov/biosample/docs/antibiogram/). Even more importantly, clinical phenotypes (including relevant outcomes and relevant treatment and confounder factors) should be mapped from carefully designed, prospective cohorts [90]. This integrative approach combining publicly available databases, curated microbiological and clinical phenotypes and powerful computational tools will pave the way for bacterial genomics to move from population studies to patient management.

## Data bibliography

1. Petit RA III, Read TD. *Staphylococcus aureus* viewed from the perspective of 40,000+ genomes. *PeerJ* 2018;6:e5261. doi.org/10.7717/peerj.5261 (2018).

## References

[1] Wertheim HFL, Melles DC, Vos MC, van Leeuwen W, van Belkum A (2005). The role of nasal carriage in *Staphylococcus aureus* infections. Lancet Infect Dis.

[2] Tong SYC, Davis JS, Eichenberger E, Holland TL, Fowler VG (2015). *Staphylococcus aureus* infections: epidemiology, pathophysiology, clinical manifestations, and management. Clin Microbiol Rev.

[3] Miro JM, Anguera I, Cabell CH, Chen AY, Stafford JA (2005). *Staphylococcus aureus* native valve infective endocarditis: report of 566 episodes from the International Collaboration on Endocarditis Merged Database. Clin Infect Dis.

[4] Fowler VG, Miro JM, Hoen B, Cabell CH, Abrutyn E (2005). *Staphylococcus aureus* endocarditis: a consequence of medical progress. JAMA.

[5] Self WH, Wunderink RG, Williams DJ, Zhu Y, Anderson EJ (2016). *Staphylococcus aureus* community-acquired pneumonia: prevalence, clinical characteristics, and outcomes. Clin Infect Dis.

[6] Holmes NE, Robinson JO, van Hal SJ, Munckhof WJ, Athan E (2018). Morbidity from in-hospital complications is greater than treatment failure in patients with *Staphylococcus aureus* bacteraemia. BMC Infect Dis.

[7] Barber M, Rozwadowska-Dowzenko M (1948). Infection by penicillin-resistant staphylococci. The Lancet.

[8] Jessen O, Rosendal K, Bülow P, Faber V, Eriksen KR (1969). Changing staphylococci and staphylococcal infections. A ten-year study of bacteria and cases of bacteremia. N Engl J Med.

[9] Chambers HF, Deleo FR (2009). Waves of resistance: *Staphylococcus aureus* in the antibiotic era. Nat Rev Microbiol.

[10] Barber M (1961). Methicillin-resistant staphylococci. J Clin Pathol.

[11] Harkins CP, Pichon B, Doumith M, Parkhill J, Westh H (2017). Methicillin-resistant *Staphylococcus aureus* emerged long before the introduction of methicillin into clinical practice. Genome Biol.

[12] Hiramatsu K, Hanaki H, Ino T, Yabuta K, Oguri T (1997). Methicillin-resistant *Staphylococcus aureus* clinical strain with reduced vancomycin susceptibility. J Antimicrob Chemother.

[13] Moellering RC (2006). Vancomycin: a 50-year reassessment. Clin Infect Dis.

[14] Fowler VG, Boucher HW, Corey GR, Abrutyn E, Karchmer AW (2006). Daptomycin versus standard therapy for bacteremia and endocarditis caused by *Staphylococcus aureus*. N Engl J Med.

[15] Mendes RE, Hogan PA, Jones RN, Sader HS, Flamm RK (2016). Surveillance for linezolid resistance via the Zyvox® annual appraisal of potency and spectrum (ZAAPS) programme (2014): evolving resistance mechanisms with stable susceptibility rates. J Antimicrob Chemother.

[16] Hartman BJ, Tomasz A (1984). Low-affinity penicillin-binding protein associated with beta-lactam resistance in *Staphylococcus aureus*. J Bacteriol.

[17] Zhang HZ, Hackbarth CJ, Chansky KM, Chambers HF (2001). A proteolytic transmembrane signaling pathway and resistance to beta-lactams in staphylococci. Science.

[18] Matthews PR, Stewart PR (1984). Resistance heterogeneity in methicillin-resistant *Staphylococcus aureus*. FEMS Microbiol Lett.

[19] Aedo S, Tomasz A (2016). Role of the stringent stress response in the antibiotic resistance phenotype of methicillin-resistant *Staphylococcus aureus*. Antimicrob Agents Chemother.

[20] Gaca AO, Colomer-Winter C, Lemos JA (2015). Many means to a common end: the intricacies of (p)ppGpp metabolism and its control of bacterial homeostasis. J Bacteriol.

[21] Pardos de la Gandara M, Borges V, Chung M, Milheiriço C, Gomes JP (2018). Genetic determinants of high-level oxacillin resistance in methicillin-resistant *Staphylococcus aureus*. Antimicrob Agents Chemother.

[22] Berger-Bächi B, Barberis-Maino L, Strässle A, Kayser FH, FemA KFH (1989). Fema, a host-mediated factor essential for methicillin resistance in *Staphylococcus aureus*: molecular cloning and characterization. Mol Gen Genet.

[23] García-Álvarez L, Holden MTG, Lindsay H, Webb CR, Brown DFJ (2011). Meticillin-resistant *Staphylococcus aureus* with a novel mecA homologue in human and bovine populations in the UK and Denmark: a descriptive study. Lancet Infect Dis.

[24] Paterson GK, Harrison EM, Holmes MA (2014). The emergence of mecC methicillin-resistant *Staphylococcus aureus*. Trends Microbiol.

[25] Diaz R, Ramalheira E, Afreixo V, Gago B (2016). Methicillin-resistant *Staphylococcus aureus* carrying the new mecC gene – a meta-analysis. Diagn Microbiol Infect Dis.

[26] Worthing KA, Coombs GW, Pang S, Abraham S, Saputra S (2016). Isolation of *mecC* MRSA in Australia. J Antimicrob Chemother.

[27] Kim CK, Milheiriço C, de Lencastre H, Tomasz A (2017). Antibiotic resistance as a stress response: recovery of high-level oxacillin resistance in methicillin-resistant *Staphylococcus aureus* “auxiliary” (*fem*) mutants by induction of the stringent stress response. Antimicrob Agents Chemother.

[28] Ba X, Harrison EM, Lovering AL, Gleadall N, Zadoks R (2015). Old drugs to treat resistant bugs: methicillin-resistant *Staphylococcus aureus* isolates with mecC are susceptible to a combination of penicillin and clavulanic acid. Antimicrob Agents Chemother.

[29] Mancini S, Laurent F, Veloso TR, Giddey M, Vouillamoz J (2015). *In* vivo effect of flucloxacillin in experimental endocarditis caused by *mecC*-positive *Staphylococcus aureus* showing temperature-dependent susceptibility *in* vitro. Antimicrob Agents Chemother.

[30] Ito T, Hiramatsu K, Tomasz A, de Lencastre H, Perreten V (2012). Guidelines for reporting novel *mecA* gene homologues. Antimicrob Agents Chemother.

[31] Schwendener S, Cotting K, Perreten V (2017). Novel methicillin resistance gene mecD in clinical *Macrococcus caseolyticus* strains from bovine and canine sources. Sci Rep.

[32] Rolo J, Worning P, Nielsen JB, Bowden R, Bouchami O (2017). Evolutionary origin of the staphylococcal cassette chromosome *mec* (SCC *mec*). Antimicrob Agents Chemother.

[33] International Working Group on the Classification of Staphylococcal Cassette Chromosome Elements (IWG-SCC) (2009). Classification of staphylococcal cassette chromosome mec (SCCmec): guidelines for reporting novel SCCmec elements. Antimicrob Agents Chemother.

[34] Baines SL, Howden BP, Heffernan H, Stinear TP, Carter GP (2016). Rapid emergence and evolution of *Staphylococcus aureus* clones harboring *fusC*-containing staphylococcal cassette chromosome elements. Antimicrob Agents Chemother.

[35] Ellington MJ, Reuter S, Harris SR, Holden MTG, Cartwright EJ (2015). Emergent and evolving antimicrobial resistance cassettes in community-associated fusidic acid and meticillin-resistant *Staphylococcus aureus*. Int J Antimicrob Agents.

[36] Harris TM, Bowen AC, Holt DC, Sarovich DS, Stevens K (2018). Investigation of trimethoprim/sulfamethoxazole resistance in an emerging sequence type 5 methicillin-resistant *Staphylococcus aureus* clone reveals discrepant resistance reporting. Clin Microbiol Infect.

[37] Kaya H, Hasman H, Larsen J, Stegger M, Johannesen TB (2018). SCCmecFinder, a web-based tool for typing of staphylococcal cassette chromosome *mec* in *Staphylococcus aureus* using whole-genome sequence data. mSphere.

[38] Firth N, Jensen SO, Kwong SM, Skurray RA, Ramsay JP (2018). Staphylococcal plasmids, transposable and integrative elements. Microbiol Spectr.

[39] Xia G, Wolz C (2014). Phages of *Staphylococcus aureus* and their impact on host evolution. Infect Genet Evol.

[40] Jones D, Elshaboury RH, Munson E, Dilworth TJ (2018). A retrospective analysis of treatment and clinical outcomes among patients with methicillin-susceptible *Staphylococcus aureus* bloodstream isolates possessing detectable *mecA* by a commercial PCR assay compared to patients with methicillin-resistant *Staphylococcus aureus* bloodstream isolates. Antimicrob Agents Chemother.

[41] Proulx MK, Palace SG, Gandra S, Torres B, Weir S (2016). Reversion from methicillin susceptibility to methicillin resistance in *Staphylococcus aureus* during treatment of bacteremia. J Infect Dis.

[42] Chung M, Kim CK, Conceição T, Aires-De-Sousa M, de Lencastre H (2016). Heterogeneous oxacillin-resistant phenotypes and production of PBP2a by oxacillin-susceptible/*mecA*-positive MRSA strains from Africa. J Antimicrob Chemother.

[43] Harrison EM, Ba X, Coll F, Blane B, Restif O (2019). Genomic identification of cryptic susceptibility to penicillins and β-lactamase inhibitors in methicillin-resistant *Staphylococcus aureus*. Nat Microbiol.

[44] Skinner S, Murray M, Walus T, Karlowsky JA (2009). Failure of cloxacillin in treatment of a patient with borderline oxacillin-resistant *Staphylococcus aureus* endocarditis. J Clin Microbiol.

[45] Burd EM, Alam MT, Passalacqua KD, Kalokhe AS, Eaton ME (2014). Development of oxacillin resistance in a patient with recurrent *Staphylococcus aureus* bacteremia. J Clin Microbiol.

[46] Ba X, Harrison EM, Edwards GF, Holden MTG, Larsen AR (2014). Novel mutations in penicillin-binding protein genes in clinical *Staphylococcus aureus* isolates that are methicillin resistant on susceptibility testing, but lack the mec gene. J Antimicrob Chemother.

[47] Ba X, Kalmar L, Hadjirin NF, Kerschner H, Apfalter P (2019). Truncation of GdpP mediates β-lactam resistance in clinical isolates of *Staphylococcus aureus*. J Antimicrob Chemother.

[48] Argudín MA, Roisin S, Nienhaus L, Dodémont M, de Mendonça R (2018). Genetic diversity among *Staphylococcus aureus* isolates showing oxacillin and/or cefoxitin resistance not linked to the presence of *mec* genes. Antimicrob Agents Chemother.

[49] Corrigan RM, Gründling A (2013). Cyclic di-AMP: another second messenger enters the fray. Nat Rev Microbiol.

[50] Planet PJ (2017). Life after USA300: the rise and fall of a superbug. J Infect Dis.

[51] Turner NA, Sharma-Kuinkel BK, Maskarinec SA, Eichenberger EM, Shah PP (2019). Methicillin-resistant *Staphylococcus aureus*: an overview of basic and clinical research. Nat Rev Microbiol.

[52] Grundmann H, Aanensen DM, van den Wijngaard CC, Spratt BG, Harmsen D (2010). Geographic distribution of *Staphylococcus aureus* causing invasive infections in Europe: a molecular-epidemiological analysis. PLoS Med.

[53] Harris SR, Feil EJ, Holden MTG, Quail MA, Nickerson EK (2010). Evolution of MRSA during hospital transmission and intercontinental spread. Science.

[54] Köser CU, Holden MTG, Ellington MJ, Cartwright EJP, Brown NM (2012). Rapid whole-genome sequencing for investigation of a neonatal MRSA outbreak. N Engl J Med.

[55] Senn L, Clerc O, Zanetti G, Basset P, Prod'hom G (2016). The stealthy superbug: the role of asymptomatic enteric carriage in maintaining a long-term hospital outbreak of ST228 methicillin-resistant *Staphylococcus aureus*. mBio.

[56] Harris SR, Cartwright EJP, Török ME, Holden MTG, Brown NM (2013). Whole-genome sequencing for analysis of an outbreak of meticillin-resistant *Staphylococcus aureus*: a descriptive study. Lancet Infect Dis.

[57] Uhlemann A-C, Dordel J, Knox JR, Raven KE, Parkhill J (2014). Molecular tracing of the emergence, diversification, and transmission of *S. aureus* sequence type 8 in a New York community. Proc Natl Acad Sci USA.

[58] Price LB, Stegger M, Hasman H, Aziz M, Larsen J (2012). *Staphylococcus aureus* CC398: host adaptation and emergence of methicillin resistance in livestock. mBio.

[59] Gonçalves da Silva A, Baines SL, Carter GP, Heffernan H, French NP (2017). A phylogenomic framework for assessing the global emergence and evolution of clonal complex 398 methicillin-resistant *Staphylococcus aureus*. Microb Genom.

[60] Aanensen DM, Feil EJ, Holden MTG, Dordel J, Yeats CA (2016). Whole-genome sequencing for routine pathogen surveillance in public health: a population snapshot of invasive *Staphylococcus aureus* in Europe. mBio.

[61] Reuter S, Török ME, Holden MTG, Reynolds R, Raven KE (2016). Building a genomic framework for prospective MRSA surveillance in the United Kingdom and the Republic of Ireland. Genome Res.

[62] Toleman MS, Reuter S, Jamrozy D, Wilson HJ, Blane B (2019). Prospective genomic surveillance of methicillin-resistant Staphylococcus aureus (MRSA) associated with bloodstream infection, England, 1 October 2012 to 30 September 2013. Euro Surveill.

[63] Price JR, Cole K, Bexley A, Kostiou V, Eyre DW (2017). Transmission of *Staphylococcus aureus* between health-care workers, the environment, and patients in an intensive care unit: a longitudinal cohort study based on whole-genome sequencing. Lancet Infect Dis.

[64] Coll F, Harrison EM, Toleman MS, Reuter S, Raven KE (2017). Longitudinal genomic surveillance of MRSA in the UK reveals transmission patterns in hospitals and the community. Sci Transl Med.

[65] Petit RA, Read TD (2018). *Staphylococcus aureus* viewed from the perspective of 40,000+ genomes. PeerJ.

[66] Guérillot R, Gonçalves da Silva A, Monk I, Giulieri S, Tomita T (2018). Convergent evolution driven by rifampin exacerbates the global burden of drug-resistant *Staphylococcus aureus*. mSphere.

[67] Bradley P, den Bakker HC, Rocha EPC, McVean G, Iqbal Z (2019). Ultrafast search of all deposited bacterial and viral genomic data. Nat Biotechnol.

[68] Copin R, Shopsin B, Torres VJ (2018). After the deluge: mining *Staphylococcus aureus* genomic data for clinical associations and host-pathogen interactions. Curr Opin Microbiol.

[69] Holden MTG, Hsu L-Y, Kurt K, Weinert LA, Mather AE (2013). A genomic portrait of the emergence, evolution, and global spread of a methicillin-resistant *Staphylococcus aureus* pandemic. Genome Res.

[70] Baines SL, Holt KE, Schultz MB, Seemann T, Howden BO (2015). Convergent adaptation in the dominant global hospital clone ST239 of methicillin-resistant *Staphylococcus aureus*. mBio.

[71] Stinear TP, Holt KE, Chua K, Stepnell J, Tuck KL (2014). Adaptive change inferred from genomic population analysis of the ST93 epidemic clone of community-associated methicillin-resistant *Staphylococcus aureus*. Genome Biol Evol.

[72] Copin R, Sause WE, Fulmer Y, Balasubramanian D, Dyzenhaus S (2019). Sequential evolution of virulence and resistance during clonal spread of community-acquired methicillin-resistant *Staphylococcus aureus*. Proc Natl Acad Sci USA.

[73] van Hal SJ, Steinig EJ, Andersson P, Holden MTG, Harris SR (2018). Global scale dissemination of ST93: a divergent *Staphylococcus aureus* epidemic lineage that has recently emerged from remote Northern Australia. Front Microbiol.

[74] DeLeo FR, Kennedy AD, Chen L, Bubeck Wardenburg J, Kobayashi SD (2011). Molecular differentiation of historic phage-type 80/81 and contemporary epidemic *Staphylococcus aureus*. Proc Natl Acad Sci USA.

[75] Tong SYC, Holden MTG, Nickerson EK, Cooper BS, Köser CU (2015). Genome sequencing defines phylogeny and spread of methicillin-resistant *Staphylococcus aureus* in a high transmission setting. Genome Res.

[76] Ward MJ, Goncheva M, Richardson E, McAdam PR, Raftis E (2016). Identification of source and sink populations for the emergence and global spread of the East-Asia clone of community-associated MRSA. Genome Biol.

[77] Lindsay JA (2013). Hospital-associated MRSA and antibiotic resistance – what have we learned from genomics?. Int J Med Microbiol.

[78] Couderc C, Jolivet S, Thiébaut ACM, Ligier C, Remy L (2014). Fluoroquinolone use is a risk factor for methicillin-resistant *Staphylococcus aureus* acquisition in long-term care facilities: a nested case-case-control study. Clin Infect Dis.

[79] Dweba CC, Zishiri OT, El Zowalaty ME (2018). Methicillin-resistant *Staphylococcus aureus*: livestock-associated, antimicrobial, and heavy metal resistance. Infect Drug Resist.

[80] Williamson DA, Carter GP, Howden BP (2017). Current and emerging topical antibacterials and antiseptics: agents, action, and resistance patterns. Clin Microbiol Rev.

[81] Ortwine JK, Werth BJ, Sakoulas G, Rybak MJ (2013). Reduced glycopeptide and lipopeptide susceptibility in *Staphylococcus aureus* and the "seesaw effect": Taking advantage of the back door left open?. Drug Resist Updat.

[82] Liu J, Chen D, Peters BM, Li L, Li B (2016). Staphylococcal chromosomal cassettes mec (SCCmec): a mobile genetic element in methicillin-resistant *Staphylococcus aureus*. Microb Pathog.

[83] Byrne ME, Gillespie MT, Skurray RA (1990). Molecular analysis of a gentamicin resistance transposonlike element on plasmids isolated from North American *Staphylococcus aureus* strains. Antimicrob Agents Chemother.

[84] Carter GP, Schultz MB, Baines SL, Gonçalves da Silva A, Heffernan H (2018). Topical antibiotic use coselects for the carriage of mobile genetic elements conferring resistance to unrelated antimicrobials in *Staphylococcus aureus*. Antimicrob Agents Chemother.

[85] Schmitz FJ, Jones ME, Hofmann B, Hansen B, Scheuring S (1998). Characterization of grlA, grlB, gyrA, and gyrB mutations in 116 unrelated isolates of *Staphylococcus aureus* and effects of mutations on ciprofloxacin MIC. Antimicrob Agents Chemother.

[86] Baines SL, Jensen SO, Firth N, Gonçalves da Silva A, Seemann T (2019). Remodeling of pSK1 family plasmids and enhanced chlorhexidine tolerance in a dominant hospital lineage of methicillin-resistant *Staphylococcus aureus*. Antimicrob Agents Chemother.

[87] Patel JB, Gorwitz RJ, Jernigan JA (2009). Mupirocin resistance. Clin Infect Dis.

[88] Yokoyama M, Stevens E, Laabei M, Bacon L, Heesom K (2018). Epistasis analysis uncovers hidden antibiotic resistance-associated fitness costs hampering the evolution of MRSA. Genome Biol.

[89] Laabei M, Massey R (2016). Using functional genomics to decipher the complexity of microbial pathogenicity. Curr Genet.

[90] Giulieri SG, Holmes NE, Stinear TP, Howden BP (2016). Use of bacterial whole-genome sequencing to understand and improve the management of invasive *Staphylococcus aureus* infections. Expert Rev Anti Infect Ther.

[91] Bergdoll M, Crass BA, Reiser RF, Robbins RN, Davis JP (1981). A new staphylococcal enterotoxin, enterotoxin F, associated with toxic-shock-syndrome *Staphylococcus aureus* isolates. The Lancet.

[92] Gillet Y, Henry T, Vandenesch F (2018). Fulminant staphylococcal infections. Microb Spectr.

[93] Chua KYL, Monk IR, Lin Y-H, Seemann T, Tuck KL (2014). Hyperexpression of α-hemolysin explains enhanced virulence of sequence type 93 community-associated methicillin-resistant *Staphylococcus aureus*. BMC Microbiol.

[94] Gillet Y, Issartel B, Vanhems P, Fournet J-C, Lina G (2002). Association between *Staphylococcus aureus* strains carrying gene for Panton-Valentine leukocidin and highly lethal necrotising pneumonia in young immunocompetent patients. The Lancet.

[95] Laabei M, Uhlemann A-C, Lowy FD, Austin ED, Yokoyama M (2015). Evolutionary trade-offs underlie the multi-faceted virulence of *Staphylococcus aureus*. PLoS Biol.

[96] Vandenesch F, Lina G, Henry T (2012). *Staphylococcus aureus* hemolysins, Bi-component leukocidins, and cytolytic peptides: a redundant arsenal of membrane-damaging virulence factors?. Front Cell Infect Microbiol.

[97] Otto M (2013). Community-Associated MRSA: what makes them special?. Int J Med Microbiol.

[98] Young BC, Earle SG, Soeng S, Sar P, Kumar V (2019). Panton–Valentine leucocidin is the key determinant of *Staphylococcus aureus* pyomyositis in a bacterial GWAS. eLife.

[99] Rudkin JK, Laabei M, Edwards AM, Joo H-S, Otto M (2014). Oxacillin alters the toxin expression profile of community-associated methicillin-resistant *Staphylococcus aureus*. Antimicrob Agents Chemother.

[100] David MZ, Daum RS (2010). Community-associated methicillin-resistant *Staphylococcus aureus*: epidemiology and clinical consequences of an emerging epidemic. Clin Microbiol Rev.

[101] Li M, Du X, Villaruz AE, Diep BA, Wang D (2012). MRSA epidemic linked to a quickly spreading colonization and virulence determinant. Nat Med.

[102] Levine DP (2006). Vancomycin: a history. Clin Infect Dis.

[103] Lee JYH, Howden BP (2015). Vancomycin in the treatment of methicillin-resistant *Staphylococcus aureus* – a clinician’s guide to the science informing current practice. Expert Rev Anti Infect Ther.

[104] Weigel LM, Clewell DB, Gill SR, Clark NC, McDougal LK (2003). Genetic analysis of a high-level vancomycin-resistant isolate of *Staphylococcus aureus*. Science.

[105] Périchon B, Courvalin P (2009). VanA-type vancomycin-resistant *Staphylococcus aureus*. Antimicrob Agents Chemother.

[106] Chang S, Sievert DM, Hageman JC, Boulton ML, Tenover FC (2003). Infection with vancomycin-resistant *Staphylococcus aureus* containing the *vanA* resistance gene. N Engl J Med.

[107] Noble WC, Virani Z, Cree RG (1992). Co-transfer of vancomycin and other resistance genes from *Enterococcus faecalis* NCTC 12201 to *Staphylococcus aureus*. FEMS Microbiol Lett.

[108] McGuinness WA, Malachowa N, DeLeo FR (2017). Vancomycin resistance in *Staphylococcus aureus*. Yale J Biol Med.

[109] Ghahremani M, Jazani NH, Sharifi Y (2018). Emergence of vancomycin-intermediate and -resistant *Staphylococcus aureus* among methicillin-resistant *S. aureus* isolated from clinical specimens in the northwest of Iran. J Glob Antimicrob Resist.

[110] Kumar M (2016). Multidrug-Resistant *Staphylococcus aureus*, India, 2013-2015. Emerg Infect Dis.

[111] Kos VN, Desjardins CA, Griggs A, Cerqueira G, Van Tonder A (2012). Comparative genomics of vancomycin-resistant *Staphylococcus aureus* strains and their positions within the clade most commonly associated with methicillin-resistant *S. aureus* hospital-acquired infection in the United States. mBio.

[112] Howden BP, Johnson PDR, Ward PB, Stinear TP, Davies JK (2006). Isolates with low-level vancomycin resistance associated with persistent methicillin-resistant *Staphylococcus aureus* bacteremia. Antimicrob Agents Chemother.

[113] Pfeltz RF, Singh VK, Schmidt JL, Batten MA, Baranyk CS (2000). Characterization of passage-selected vancomycin-resistant *Staphylococcus aureus* strains of diverse parental backgrounds. Antimicrob Agents Chemother.

[114] Howden BP, Davies JK, Johnson PDR, Stinear TP, Grayson ML (2010). Reduced vancomycin susceptibility in *Staphylococcus aureus*, including vancomycin-intermediate and heterogeneous vancomycin-intermediate strains: resistance mechanisms, laboratory detection, and clinical implications. Clin Microbiol Rev.

[115] Mwangi MM, Wu SW, Zhou Y, Sieradzki K, de Lencastre H (2007). Tracking the in vivo evolution of multidrug resistance in *Staphylococcus aureus* by whole-genome sequencing. Proc Natl Acad Sci USA.

[116] Cui L, Neoh H-M, Shoji M, Hiramatsu K (2009). Contribution of vraSR and graSR point mutations to vancomycin resistance in vancomycin-intermediate *Staphylococcus aureus*. Antimicrob Agents Chemother.

[117] Howden BP, McEvoy CRE, Allen DL, Chua K, Gao W (2011). Evolution of multidrug resistance during *Staphylococcus aureus* infection involves mutation of the essential two component regulator WalKR. PLoS Pathog.

[118] Cui L, Isii T, Fukuda M, Ochiai T, Neoh H-M (2010). An rpoB mutation confers dual heteroresistance to daptomycin and vancomycin in *Staphylococcus aureus*. Antimicrob Agents Chemother.

[119] Passalacqua KD, Satola SW, Crispell EK, Read TD (2012). A mutation in the PP2C phosphatase gene in a *Staphylococcus aureus* USA300 clinical isolate with reduced susceptibility to vancomycin and daptomycin. Antimicrob Agents Chemother.

[120] Giulieri SG, Baines SL, Guerillot R, Seemann T, Gonçalves da Silva A (2018). Genomic exploration of sequential clinical isolates reveals a distinctive molecular signature of persistent *Staphylococcus aureus* bacteraemia. Genome Med.

[121] McEvoy CRE, Tsuji B, Gao W, Seemann T, Porter JL (2013). Decreased vancomycin susceptibility in *Staphylococcus aureus* caused by IS*256* tempering of WalKR expression. Antimicrob Agents Chemother.

[122] Alam MT, Petit RA, Crispell EK, Thornton TA, Conneely KN (2014). Dissecting vancomycin-intermediate resistance in *Staphylococcus aureus* using genome-wide association. Genome Biol Evol.

[123] Rishishwar L, Petit RA, Kraft CS, Jordan IK (2014). Genome sequence-based discriminator for vancomycin-intermediate *Staphylococcus aureus*. J Bacteriol.

[124] Zhang S, Sun X, Chang W, Dai Y, Ma X (2015). Systematic review and meta-analysis of the epidemiology of vancomycin-intermediate and heterogeneous vancomycin-intermediate *Staphylococcus aureus* isolates. PLoS One.

[125] Holmes NE, Turnidge JD, Munckhof WJ, Robinson JO, Korman TM (2014). Genetic and molecular predictors of high vancomycin MIC in *Staphylococcus aureus* bacteremia isolates. J Clin Microbiol.

[126] Howden BP, Peleg AY, Stinear TP (2014). The evolution of vancomycin intermediate *Staphylococcus aureus* (VISA) and heterogenous-VISA. Infect Genet Evol.

[127] Gardete S, Tomasz A (2014). Mechanisms of vancomycin resistance in *Staphylococcus aureus*. J Clin Invest.

[128] Humphries RM, Pollett S, Sakoulas G (2013). A current perspective on daptomycin for the clinical microbiologist. Clin Microbiol Rev.

[129] Kelley PG, Gao W, Ward PB, Howden BP (2011). Daptomycin non-susceptibility in vancomycin-intermediate *Staphylococcus aureus* (VISA) and heterogeneous-VISA (hVISA): implications for therapy after vancomycin treatment failure. J Antimicrob Chemother.

[130] Sharma M, Riederer K, Chase P, Khatib R (2008). High rate of decreasing daptomycin susceptibility during the treatment of persistent *Staphylococcus aureus* bacteremia. Eur J Clin Microbiol Infect Dis.

[131] Bayer AS, Schneider T, Sahl H-G (2013). Mechanisms of daptomycin resistance in *Staphylococcus aureus*: role of the cell membrane and cell wall. Ann NY Acad Sci.

[132] Yang S-J, Mishra NN, Rubio A, Bayer AS (2013). Causal role of single nucleotide polymorphisms within the *mprF* gene of *Staphylococcus aureus* in daptomycin resistance. Antimicrob Agents Chemother.

[133] Friedman L, Alder JD, Silverman JA (2006). Genetic changes that correlate with reduced susceptibility to daptomycin in *Staphylococcus aureus*. Antimicrob Agents Chemother.

[134] Adhikari RP (2004). Vancomycin-induced deletion of the methicillin resistance gene mecA in *Staphylococcus aureus*. J Antimicrob Chemother.

[135] Renzoni A, Kelley WL, Rosato RR, Martinez MP, Roch M (2017). Molecular bases determining daptomycin resistance-mediated resensitization to β-lactams (seesaw effect) in methicillin-resistant *Staphylococcus aureus*. Antimicrob Agents Chemother.

[136] Wang G, Hindler JF, Ward KW, Bruckner DA (2006). Increased vancomycin MICs for *Staphylococcus aureus* clinical isolates from a university hospital during a 5-year period. J Clin Microbiol.

[137] Locke JB, Hilgers M, Shaw KJ (2009). Novel ribosomal mutations in *Staphylococcus aureus* strains identified through selection with the oxazolidinones linezolid and torezolid (TR-700). Antimicrob Agents Chemother.

[138] Locke JB, Rahawi S, LaMarre J, Mankin AS, Shaw KJ (2012). Genetic environment and stability of *cfr* in methicillin-resistant *Staphylococcus aureus* CM05. Antimicrob Agents Chemother.

[139] Locke JB, Zuill DE, Scharn CR, Deane J, Sahm DF (2014). Identification and characterization of linezolid-resistant *cfr*-positive *Staphylococcus aureus* USA300 isolates from a New York City medical center. Antimicrob Agents Chemother.

[140] Sánchez García M, De la Torre MA, Morales G, Peláez B, José Tolón M (2010). Clinical outbreak of linezolid-resistant *Staphylococcus aureus* in an intensive care unit. JAMA.

[141] Geriak M, Haddad F, Rizvi K, Rose W, Kullar R (2019). Clinical data on daptomycin plus ceftaroline versus standard of care monotherapy in the treatment of methicillin-resistant *Staphylococcus aureus* bacteremia. Antimicrob Agents Chemother.

[142] Long SW, Olsen RJ, Mehta SC, Palzkill T, Cernoch PL (2014). Pbp2A mutations causing high-level ceftaroline resistance in clinical methicillin-resistant *Staphylococcus aureus* isolates. Antimicrob Agents Chemother.

[143] Wüthrich D, Cuénod A, Hinic V, Morgenstern M, Khanna N (2019). Genomic characterization of inpatient evolution of MRSA resistant to daptomycin, vancomycin and ceftaroline. J Antimicrob Chemother.

[144] Alm RA, McLaughlin RE, Kos VN, Sader HS, Iaconis JP (2014). Analysis of *Staphylococcus aureus* clinical isolates with reduced susceptibility to ceftaroline: an epidemiological and structural perspective. J Antimicrob Chemother.

[145] Lee H, Yoon E-J, Kim D, Kim JW, Lee K-J (2018). Ceftaroline resistance by clone-specific polymorphism in penicillin-binding protein 2a of methicillin-resistant *Staphylococcus aureus*. Antimicrob Agents Chemother.

[146] Abbott IJ, Jenney AWJ, Jeremiah CJ, Mirčeta M, Kandiah JP (2015). Reduced *in vitro* activity of ceftaroline by Etest among clonal complex 239 methicillin-resistant *Staphylococcus aureus* clinical strains from Australia. Antimicrob Agents Chemother.

[147] Bradley P, Gordon NC, Walker TM, Dunn L, Heys S (2015). Rapid antibiotic-resistance predictions from genome sequence data for *Staphylococcus aureus* and *Mycobacterium tuberculosis*. Nat Commun.

[148] Gordon NC, Price JR, Cole K, Everitt R, Morgan M (2014). Prediction of *Staphylococcus aureus* antimicrobial resistance by whole-genome sequencing. J Clin Microbiol.

[149] Williamson DA, Heffernan H, Nimmo G (2015). Contemporary genomic approaches in the diagnosis and typing of *Staphylococcus aureus*. Pathology.

[150] Lower SK, Lamlertthon S, Casillas-Ituarte NN, Lins RD, Yongsunthon R (2011). Polymorphisms in fibronectin binding protein A of *Staphylococcus aureus* are associated with infection of cardiovascular devices. Proc Natl Acad Sci USA.

[151] Recker M, Laabei M, Toleman MS, Reuter S, Saunderson RB (2017). Clonal differences in *Staphylococcus aureus* bacteraemia-associated mortality. Nat Microbiol.

[152] Lilje B, Rasmussen RV, Dahl A, Stegger M, Skov RL (2017). Whole-genome sequencing of bloodstream *Staphylococcus aureus* isolates does not distinguish bacteraemia from endocarditis. Microb Genom.

[153] Read TD, Massey RC (2014). Characterizing the genetic basis of bacterial phenotypes using genome-wide association studies: a new direction for bacteriology. Genome Med.

[154] Scott WK, Medie FM, Ruffin F, Sharma-Kuinkel BK, Cyr DD (2018). Human genetic variation in GLS2 is associated with development of complicated *Staphylococcus aureus* bacteremia. PLoS Genet.

[155] Didelot X, Walker AS, Peto TE, Crook DW, Wilson DJ (2016). Within-host evolution of bacterial pathogens. Nat Rev Microbiol.

[156] Young BC, Wu C-H, Gordon NC, Cole K, Price JR (2017). Severe infections emerge from commensal bacteria by adaptive evolution. eLife.

[157] Guérillot R, Li L, Baines S, Howden B, Schultz MB (2018). Comprehensive antibiotic-linked mutation assessment by resistance mutation sequencing (RM-seq). Genome Med.

[158] Davis JS, Sud A, O'Sullivan MVN, Robinson JO, Ferguson PE (2016). Combination of vancomycin and β-lactam therapy for methicillin-resistant *Staphylococcus aureus* bacteremia: a pilot multicenter randomized controlled trial. Clin Infect Dis.

[159] Steinig EJ, Duchene S, Robinson DA, Monecke S, Yokoyama M (2019). Evolution and global transmission of a multidrug-resistant, community-associated methicillin-resistant *Staphylococcus aureus* lineage from the Indian subcontinent. mBio.

